# Tissue-specific metabolism and TRIM24

**DOI:** 10.18632/aging.100822

**Published:** 2015-10-02

**Authors:** Kaushik N. Thakkar, Sabrina A. Stratton, Michelle Craig Barton

**Affiliations:** Department of Epigenetics and Molecular Carcinogenesis, The University of Texas MD Anderson Cancer Center, Houston, TX 77030, USA

**Keywords:** epigenetics, histone reader, breast cancer, hepatocellular carcinoma

Epigenetic regulation of metabolism is critical to maintain cellular homeostasis in response to cellular demands and resources. Changes in the microenvironment impact functions of epigenetic regulatory enzymes and metabolic balance must maintained to avoid metabolic reprogramming and tumor progression [1]. Epigenetic regulatory proteins with catalytic functions include histone “writers” or “erasers”, which effect post-translational modifications (PTMs) of histones or non-histone proteins; chromatin remodelers, fueled by ATP to alter chromatin structure; and, enzymes that methylate DNA or reverse DNA methylation. Genes encoding key factors in metabolic signaling pathways are regulated by the activities of these enzymes, which remodel or modify chromatin. Additionally, epigenetic regulatory enzymes themselves serve as environmental sensors by relying on available metabolites as cofactors. For example, mutations in IDH1/IDH2, which encode isocitrate dehydrogenases of the tricarboxylic acid (TCA) cycle, are associated with low-grade gliomas, adult de novo acute myeloid leukemias and lymphomas. Mutant IDH1/2 exhibit gain-of-function by catalyzing conversion of α-ketoglutarate (α-KG) to 2-hydroxy glutarate (2-HG) at 100-fold higher levels than found in normal cells. 2-HG is a competitive inhibitor of α-KG-dependent dioxygenases, including TET2, which acts in reversal of DNA methylation, and Jumonji-C-domain-containing histone demethylases, which alter histone PTMS; either of which may disrupt regulated gene expression [2].

In addition to epigenetic regulatory enzymes that act as sensors of cellular metabolites and/or directly alter chromatin structure of genes that encode metabolic enzymes, proteins known as “histone readers” serve as relay switches in regulatory networks, including metabolism. Histone readers have specific domains that bind defined “signatures” of histone PTMs and act as platforms for recruitment of transcription factors, mediators or additional epigenetic response factors to chromatin. Our laboratory showed that histone reader Tripartite motif-containing protein 24 (TRIM24) not only negatively regulates p53 as an E3-ubiquitin ligase [3] but also interacts with and recruits transcription factors, such as estrogen receptor, to chromatin via a tandem plant homeodomain (PHD) and Bromodomain that binds unmethylated lysine 4 and acetylated lysine 23 of histone H3 (H3K4me0/H3K23ac) [4]. More recently, we found that ectopic expression of TRIM24 promoted oncogenic transformation of immortalized human mammary epithelial cells (TRIM24-iHMECs) and efficient growth of intermediate to high-grade xenograft tumors [5]. Molecular analysis of TRIM24-iHMECs revealed a TRIM24-dependent glycolytic and TCA cycle gene expression signature, which led to increased glucose uptake. Gene Set Enrichment Analysis revealed the glucose transport pathway as one of the top 10 pathways positively correlated with TRIM24 expression in human breast tumors (n = 1008) from the TCGA database. Interestingly, Seahorse analysis showed both ECAR (measure of glycolysis) and OCR (measure of OXPHOS) were elevated in TRIM24-iHMECs. This is unlike a conventional Warburg effect of unrestrained, aerobic glycolysis but consistent with recent reports of cancers that exploit OXPHOS or a mixture of glycolysis and OXPHOS for energy production [6].

In addition to its functions as a histone reader, TRIM24 is an E3-ubiquitin ligase that targets p53 for protein degradation. Tumor suppressor p53 plays key roles in glycolysis, OXPHOS, glutamine metabolism, lipid metabolism and antioxidant defense to impact cellular metabolism and redox balance [7]. We surveyed both p53-positive and -negative breast cancer-derived cell lines to assess p53-dependence of TRIM24-regulated metabolic response and found that GLUT1, ACO1, IDH1 and IDH2, which encode important players in glycolysis and TCA cycle, were TRIM24-activated in MCF7, SKBR3 and MDAMB231 cells despite their varied p53 status. This may be explained by our finding that TRIM24 directly regulates these genes, as well as another important player in oncogenesis and metabolism: c-myc. Our unpublished chromatin immunoprecipitation analyses show direct binding of TRIM24 to the upstream regulatory regions of c-myc, GLUT1, IDH1 and IDH2, concomitant with activation of transcription.

Given the clinical correlates in breast cancer patients and apparent oncogenic effects on metabolism that we reported in human mammary epithelial cells, one might expect that loss of TRIM24 in mouse models would oppose tumor development. However, complete loss of Trim24, either globally or conditionally in the liver, caused spontaneous hepatic steatosis and ultimately hepatocellular carcinoma in animals fed normal chow. With loss of Trim24 expression, hepatocytes increased expression of lipase and inflammation signaling genes and repressed de novo lipogenesis, steroid and lipid metabolism and transport. The hepatic accumulation of lipids, fibrosis and infiltration of inflammatory macrophages recapitulated parameters of human nonalcoholic fatty liver disease (NAFLD) and non-obese-NASH [8]. Whether this outcome is the result of tissuespecific shifts in transcription factors that collaborate with TRIM24, an altered collection of TRIM24 target genes and/or a tissue-specific response to regulated p53 levels is unknown at this time. Clearly, additional mouse models, especially those with conditional over expression of Trim24, and manipulation of diet, along with mechanistic studies of functional domains and intersecting pathways, are needed to determine how epigenetic variables and tissue-specificity dictate metabolic reprogramming by TRIM24.
